# The Importance of a More Thorough Knowledge of the Dental Organs on the Part of Physicians, to the Successful Management of Many Diseases—and, Equally Requisite, a More Thorough Knowledge of the General System by Dentists, for Greater Skill and Success in the Practice of Their Art

**Published:** 1855-07

**Authors:** W. R. Handy


### ARTICLE II.

*The Importance of a more thorough Knowledge of the Dental Organs on the part of Physicians, to the Successful Management of many Diseases—and, equally requisite, a more thorough Knowledge of the General System by Dentists, for greater Skill and Success in the Practice of their Art.* By W. R. HANDY, M. D.

THE few remarks we may have to offer will be confined, more especially to the first branch of the above subject, viz. the "importance of a more thorough knowledge of the dental organs on the part of physicians to the successful management of many diseases,"—the second branch may claim attention at another time.

Chomel, in his General Pathology, uses the following language, "diseases are not confined in their effects to single organs, nor can they be disconnectedly studied." So true is this remark, that few physicians, if any, pretend, for a moment, to call it in question. Yet, strange to say, few physicians seem to know scarcely any thing of the diseases of the teeth, equally as little of the influence of such diseases upon the rest of the economy, and still less, as causes of irremedial mischief.

We say that such a statement does not, however, appear so strange, when we reflect upon the fact that medical students never have their attention directed to the study of the dental organs as to the rest of the organs of the body. When the professor arrives at these organs, they are generally dismissed with a hurried casual remark, as of little importance, and consequently leave an impression upon the student's mind that they have little to do in the study of disease and its treatment, hence the little importance we so generally find attached to this branch of medicine.

All medical colleges are nearly equally culpable on this point, at least they were so when we were a student, and we have yet to learn that there has been any change.

We propose presenting a few plain, brief remarks to prove such strange neglect, or if you please, indifference, to the claims of so important a class of organs in the management of many diseases, and should our humble efforts go no further than simply to awaken a greater interest in the subject, we shall feel amply rewarded for all our labor.

The physiologist does not hesitate to assert the great fundamental principle of the unity of the organism, the inseparable condition of the whole with each and each with the whole, so close in fact, that however small or insignificant the organ or organs may seem, in a physiological point of view, the individual functions of each are regarded as necessary, yet absolutely necessary to the harmony of the whole. Yet in the face of all this, when we come to the pathology of these same organs, they are denied the same claims in disease that they have in health, and will not be allowed to scarcely constitute a link in the scale of influence in that great circle of organs, which, in the language of the poet,

> "Whatever link you strike,
> Tenth or ten-thousandth,
> Breaks the chain alike!"

Chomel remarks, "it is the peculir province of physiology to treat of whatever relates to the human body in its normal condition to pathology, includes within its limits whatever relates to the same in a state of disease." In a word, pathology goes side by side with physiology—that disease is commensurate with health, and that no organ or class of organs can be refused its appropriate place and influence in estimating the character and deciding upon the treatment of any disease or complication that may arise.

As an offset, however, to a proper estimate of the dental organs by physicians and the medical profession generally, we are happy to state, that in another and kindred department, viz. the dental, they have received due consideration.

Profs. Harris and Bond, with many others in our own and foreign countries, have, and are still nobly striving to rescue the dental organs from the ignominy to which we would charitably hope, they have so long been ignorantly consigned by physicians; and have, already, in a great measure, succeeded in convincing the medical mind that these same organs deserve equal rank, in proportion to their relative influence, with all others in the preservation of health and in the causation of disease, and should hence have equal importance attached to their study and relations with the rest of the economy.

Prof. Bond, it is true, is a physician, and until recently was extensively engaged in the practice of general medicine, but he is also one of the professors in the Baltimore Dental College, and in this connection has put forth a work upon dental medicine, though designed more especially for the dental student, yet equally necessary for the medical, as clearly showing the frequency of general disturbance from dental disease, as well as dental disease from general disturbance, and consequently the absolute necessity of the study of both kinds for the successful treatment of either.

Dr. Rush, in his Medical Inquiries, uses the following language, "when we consider how often the teeth, when decayed, are exposed to irritation from hot and cold drinks, and aliments, and from pressure by mortification, and from cold air, and how intricate the connection of the mouth is with the whole system, I am disposed to believe they are often unsuspected causes of general, and particularly of nervous diseases. When we add to the list of these diseases, the morbid effects of the acid and putrid matters which are sometimes discharged from carious teeth, or from ulcers in the gums, created by them, also the influence which both have in preventing perfect mastication, and the connection of that animal function with good health, I cannot help thinking that our success in the treatment of all chronic diseases would be very much promoted by directing our inquiries into the state of the teeth in sick people, and by advising their extraction in every case in which they are decayed."

We take the following quotations from Prof. Bond's work. Hufeland places sound teeth among the signs of long life, "for good digestion, he says, good teeth are extremely necessary, and one, therefore, may consider them among the essential properties requisite for long life, and in two points of view. First, good and strong teeth are always a sign of a sound, strong constitution and good juices. Those who lose their teeth early, have, in a certain measure, taken possession of the other world, with a part of their bodies. Secondly, the teeth are a great help to digestion, and consequently to restoration."

Baglivi observes, that "persons whose teeth are in an unclean and viscid state, though daily washed, have universally a weak stomach, bad digestion, and offensive breath, headache after meals, generally bad health and low spirits. If engaged in business or study they are irritable and impatient, and are often seized with dizziness. From weakness of the stomach they are naturally somnolent, scarcely wakeful in the morning, and never satisfied with sleep."

Mr. Liston, the great surgeon of London, thus remarks, "from the presence of carious teeth, or decayed portions of teeth, many evils, both local and general, ensue, besides inflammation, and abscess. They are frequently the cause, and the sole cause, of violent and continued headaches, of glandular swellings in the neck, terminating in, or combined with abscess—of enlargement and inflammation of the tonsils, either chronic or acute—of ulceration of the tongue and lips, often assuming a malignant action from continued irritation, of painful feelings in the face, tic douloureux, pains in the tongue, jaws, &c., of disordered stomach from affection of the nerves, or from imperfect mastication, of continued constitutional irritation, which may give rise to serious diseases."

In his second edition of the Principles and Practice of Dental Surgery, Prof. Harris gives the following quotation from Dr. Darwin in reference to the agency of the teeth in producing hemicrania and ear-ache—"This disease, hemicrania, is attended with a cold skin, and hence, whatever may be the remote cause, the immediate one seems to be a want of stimulus, either of heat or distention, or some other unknown stimulus in the painful part, or in those with which it is associated. The membranes, in their natural state, are only irritable by distention—in their diseased state, they are sensible like muscular fibres.

Hence a diseased tooth may render the neighboring membranes sensible, and is frequently the cause of this disease."

Of sympathetic headache, he adds, "where it affects a small defined part on the parietal bone or one side, it is generally termed clavies hystericus, and is always, I believe, owing to a diseased dens molaris."

Such is some of the medical authority touching the importance of the subject to the practicing physician.

As practical cases seem best suited to show to the practical man with convincing clearness, that the teeth are not only important to be known and studied, but absolutely essential to be known and thoroughly studied, we propose giving a few cases by way of illustration.

It may be proper in the first place to notice some of the chief connections which the teeth as organs have with the rest of the organs of the body, so that the sympathies of the former may be the more readily recognized and traced among those of the latter.

The fifth pair of nerves, the cranial spinal nerve of Sir Charles Bell, is the great nerve of the teeth, and its three principal divisions, viz. the ophthalmic, superior and inferior maxillary nerves, form the great nervous connection with the mouth, the tongue, the palate, tonsils, uvula, the different senses, as the eye, the ear, the taste and the smell, the brain, the spinal marrow, the sympathetic nerve, and through those latter to the whole body. In reference to this latter nerve, Kirke and Paget uses the following language, "These communicating branches, which have been generally called roots or origins of the sympathetic nerve, an interchange is effected between all the spinal nerves, and the sympathetic trunks—all the ganglia also which are seated on the cerebral nerves, as the ophthalmic, otic, &c., have roots, as they are called, through which filaments of the cerebral nerves are added to their own. So that, probably, all sympathetic nerves contain some intermingled cerebral or spinal nerve fibres, and all cerebral and spinal nerves some filaments derived from the sympathetic system or from glanglia;" in a word, interchanging and communicating by this extended nervous intercourses, including the eighth pair or parvagum, with the whole body and its several parts.

But there are other connections of the teeth with the rest of the body, the mucous membrane, from whence the teeth have their origin, extends through the entire alimentary canal in one direction, and through the larynx, trachea and bronchia, in another direction, thus associating at the same time, organs the most dissimilar, and functions the most varied; by this membrane we have thus brought together the mouth, pharynx, osephagus, stomach, the small and large intestines, the collatitious viscera, the windpipe and lungs, which with the nervous system, correspond to the functions of prehension, mastication, insalivation, deglutition, chymificaton and chylification, with those of thought, speech, expression, expiration.

The teeth have also a relation with the other organs, by means of the blood vessels. The superior and inferior dental arteries, branches of the internal maxillary, which latter is a branch of the external carotid, that can be traced to the common carotid, and thence to the aorta and heart—form the means by which the teeth in common with every other part, are kept in their integrity both of structure and function.

We are now prepared more fully for the cases proposed to be noticed, as illustrating painful, protracted and unmanageable disease in different parts of the body, arising from morbid conditions of the teeth, which so long as they remained unsuspected of being the cause of all the mischief produced, just so long did every kind of treatment not only fail but aggravate the mischief; while on the other hand, when the cause of the mischief in the diseased teeth were removed, immediately the mischief subsided and health was restored.

The first case we notice, is taken from the American Journal of Medical Science. It is a case of *photophobia* or dread of light, such an exalted sensibility of the retina existed that even the smallest amount of light, such for instance as the little reflected from the bosom of a shirt, was intolerable brightness. The most eminent physicians of North Carolina attended this case, we are told, and that every sort of treatment had been fully, but unsuccessfully tried, when on the extraction of one or more teeth, all the symptoms at once subsided; the retina recovered almost instantly, its natural sensibility to impressions of light, and the general health was completely restored.

The second case was reported to me by a female friend, in which the patient had severe pain and protracted suffering in one of the lower limbs, all the ordinary means were tried in vain, when on the extraction of a tooth, the pain vanished, and the patient recovered.

In the January No. 1855 of the American Journal of Dental Science, a physician states that a lady in health had a tooth extracted; "almost immediately, he says, *paralysis* on one side of the body occurred, then stupor and death followed in a few hours," no chloroform nor ether, it was stated, was administered.

All are familiar with the fact that teething frequently throws the child into the most frightful convulsions; this is brought about by the fifth pair of nerves and the brain acting upon the voluntary muscular system.

The following cases illustrating the complete dependence of other diseases, upon disease of the teeth as their cause, are taken from Professor Harris' work upon the Principles and Practice of Dental Surgery, second edition.

"A lady of this city, and of high respectability, says the doctor, was advised by her family physician to consult me in relation to her teeth: her health at this time was very delicate and precarious, and had been so for six years. She had taken much medicine, and had visited the Saratoga and White Sulphur Springs, but without obtaining any permanent relief. Her stomach was so much disordered, that the lightest kind of food, produced, for several hours after it had been taken, a heavy, burning, and very painful sensation. Her whole nervous system was so completely deranged, that the quick slamming of a door, or any other sudden noise, would almost throw her into convulsions. Her eyesight was much impaired, and her head affected with almost constant swimming, or dizziness. On examining her mouth, I found the crowns of the superior incisors, cuspidati and bicuspids, the inferior molars, a bicuspid on the right, and a dens sapientiæ on the left side of the lower jaw, involved in general and complicated caries. Their alveolar processes were more or less wasted, the gums tumefied, soft, spongy and ulcerated along their edges. The inner surface of the inferior incisors, and the outer surface of the superior molars were thickly coated with tartar, and the salivary and mucous secretions of the mouth in a vitiated condition. At the earnest request of her friends, she submitted to the necessary dental treatment, by which the health of her mouth was completely restored, and with it that of her general system also."

In this case we see general derangement of the whole system, especially of the nerves, the stomach, and the eyes, and the whole restored by simply correcting the disease of the teeth. The next case is that of a gentleman with *dyspepsia:* he had been in bad health for four or five years, during which time he had applied to the most eminent physicians of New York, Troy and Albany, without obtaining any permanent relief. "The character of the symptoms, says the doctor, that at this time prevailed, was very peculiar. His digestive organs were so much deranged, that he was obliged to observe the strictest regimen, and confine himself to the simplest kind of vegetable food. Besides the dyspeptic affection with which he was troubled, he had severe periodical paroxysms of head-ache and vomiting, recurring at regular intervals, of from four to five weeks. These were always preceded by numbness, commencing first in his tongue, and thence extending throughout the whole system; the sensation generally continued for about two hours, when it was succeeded by violent pain in the head, and partial vertigo, from which in about ten hours he was relieved by vomiting. The effects of these paroxysms lasted about ten days, and the other symptoms had continued, without much mitigation, for three years." The teeth, as many as required removal, were extracted; all disease removed, and in "about two months, we are told, the general health was perfectly restored, and in about four or five weeks, the local affection had disappeared."

*Epilepsy* and *Rheumatism* are mentioned by Dr. Rush, as produced by decayed teeth. "Sometime in the year 1801, I was consulted by the father of a young gentleman in Baltimore, who had been affected with epilepsy. I inquired into the state of his teeth, and was informed that several of them, in his upper jaw, were much decayed. I directed them to be extracted, and advised him afterwards to lose a few ounces of blood any time when he felt the premonitory symptoms of a recurrence of his fits. He followed my advice, in consequence of which, I had lately the pleasure of hearing from his brother, that he was perfectly cured."

The case of rheumatism, was in the hip-joint of a lady. Several remedies had been tried, with only partial benefit, when, from a severe tooth-ache which accompanied the disease, the tooth was extracted; the rheumatism disappeared, and we are informed, never returned.

*Ophthalmia*, *Ear-ache*, *Head-ache*, *Phthisis*, with many other cases, if our limits allowed, might be cited to prove that diseased teeth, were not only frequently, but very frequently the unsuspected cause of numerous diseases in varous parts of the body. We will close by giving one other very interesting case from Dr. Koecker, a distinguished dentist of England. The case is one of *Neuralgia of the face*, or *tic douloureux*.

"Mr. J., says the doctor, a gentleman of great respectability, a native of this country, but for many years a resident of Smyrna, aged about thirty-nine years, had suffered upwards of ten years with this distressing malady, attended by all its torturing symptoms, in a most unparalleled manner. His whole constitution, but particularly the glandular system, was so much affected as to produce swellings and indurations in the most distant parts, accompanied with great pain and inconvenience, but its effects on his head were frequently agonizing; indeed, he assured me, so great were his sufferings, he had been so far driven to despair, as to implore heaven to relieve him, by putting an end to his miserable existence. He repeatedly applied for the best medical and surgical advice that the country could afford, but the real cause of his suffering was not detected; and such was the character of this disorder, that it baffled every exertion, and all the remedies, which were applied for many years. At length, the effects of a sea voyage, and a visit to his native country, were proposed, and at the same time, a trial of such medical measures as he might be able to command in England.

On reaching England, Dr. Lawrence was consulted, and advised his teeth to be attended to, this was done, and the report concludes, that "in less than one month he was restored to perfect constitutional health."

We trust the above facts are sufficient to satisfactorily prove the position we started with, viz. that the dental organs were the cause, and the not unfrequent cause of unsuspected disease in different parts of the body, and that unless this cause be known and removed, the physician invariably failed in curing his patient, and hence, was urged the importance, and may we not add the great importance, that every physician should study the dental organs equally with the rest of the body, that he may thereby be equally prepared to combat disease originating from this source.

To the dentist, we would beg leave to caution him of the danger of running into the other extreme, and in direct antagonism with the physician, to suppose that all diseases originate in the teeth, and that by removing the morbid conditions of the latter, that disease existing in any portion of the body, however distant, and of whatever kind, would also with equal certainty be removed.

This is a fallacy, we feel justified in saying, as much at war with every scientific principle, which should guide the truly educated and high minded dentist, as that of the physician, who, is too prone to exclude or deny to the teeth any share whatever in the production of disease, except so far as the mouth may be concerned.

The truth is, there are many diseases, whose power is spent upon the dental organs, while their source or cause is seated in different and often distinct parts—now to remove the teeth under such circumstances, would not at all remove the disease, but would rather, if any thing, have a tendency to aggravate it, and thus to expose the ignorance and hazard the reputation of the operator, for he should have so far mastered his subject, as to know that nothing short of an acquaintance with the entire body, could properly constitute a scientific dentist, and that the specialty of his art though practically confined to the mouth, nevertheless, extends its research to every organ of the body, and draws from thence its greatest skill and success, that here is the distinguishing line between the scientific dentist and the mere pretender or charlatan, between the man guided by study and principle, and the man led on by ignorance and falsehood.

To the former, his skill will save the teeth of his patient, as well as his reputation in all those cases where intermittent fever, scurvy, affections of the stomach, uterus, &c., report their violence upon the dental apparatus. While to the latter the converse is equally true, that destruction of the teeth and loss of reputation must as infallibly follow.

